# Accuracy of body composition measurements by dual energy x-ray absorptiometry in underweight patients with chronic intestinal disease and in lean subjects

**DOI:** 10.1186/1476-5918-4-1

**Published:** 2005-01-04

**Authors:** Kent Valentin Haderslev, Pernille Heldager Haderslev, Michael Staun

**Affiliations:** 1 Department of medical Gastroenterology, Copenhagen University Hospital, Rigshospitalet, Blegdamsvej 9, 2100 Copenhagen, Denmark

## Abstract

**Background:**

To assess the accuracy of Dual-energy X-ray absorptiometry (DXA) in underweight patients with chronic gastrointestinal disease, we investigated the ability of DXA to detect variations in body composition induced by infusion of parenteral nutrition (PN). Furthermore, the influence of a low body weight per se on the accuracy of DXA was studied by placing packets of lard on lean healthy subjects.

**Methods:**

The hydration study included 11 patients with short bowel syndrome on long-term home parenteral nutrition (9 women and 2 men), and (mean ± SD) 49.5 ± 17.1 yr., 19.3 ± 3.1 kg/m^2^. The lard study, where packets of lard were placed either over the thighs or the trunk region, was performed in 8 healthy lean male volunteers, 26.4 ± 7.4 yr., and 21.0 + 0.9 kg/m^2^. Body composition, including measures of the total mass (TM), soft tissue mass (STM), lean tissue mass (LTM), fat mass (FM), and total body mineral content (TBBMC), was assessed by DXA. The fat fraction of the lard packets (3.49 kg), measured in triplicate by chemical fat extraction, was 52.2%.

**Results:**

*Hydration study; *The increase in scale weight (BW) of approximately 0.90 kg due to infusion of PN correlated significantly to the increase in TM (R-square = 0.72, SEE 0.36 kg, p < 0.01), and the increase in STM (R-square = 0.69, SEE 0.38 kg, p < 0.01), however not with the increase LTM (R-square = 0.30, SEE 1.06 kg, p = 0.08). Mean changes in TM (0.88 kg), STM (0.88 kg), and LTM (0.81 kg) were not significantly different from changes in BW (p > 0.05). *Lard study; *Regardless of position, measurements of FM and LTM of the added lard were not significantly different from expected values. However, the composition of the lard packets into FM and LTM was more accurately detected when the packets were placed over the thighs than over the trunk region. The accuracy of DXA in individual subjects, expressed as the SD of the difference between expected and measured values, was 1.03 kg and 1.06 kg for the detection of changes in LTM and FM, respectively, and 0.18 kg for the detection of changes in STM and TM.

**Conclusions:**

On a group level, DXA provided sufficient accuracy to detect small changes in body composition in underweight patients with chronic gastrointestinal disease. However, the accuracy errors were higher than reported in normal weight subjects. The accuracy was not influenced by a low body weight per se.

## Background

Malnutrition is commonly observed in patients with chronic gastrointestinal disease and nutritional support is therefore an integral part of the management of these patients. Accurate and precise methods that are sensitive enough to track small changes in body composition are important for assessing the effect of nutritional intervention. At present, there are available a variety of well-established techniques such as hydrodensitometry, isotope dilution, air-displacement plethysmography [1], and potassium spectroscopy, methods which are based on the 2-compartment model separating the body into fat tissue mass (FM) and fat-free tissue mass (FFM). However, these methods rely on assumptions that are often not met in malnourished patients, e.g. a fixed hydration and density of the FFM, which may negatively influence the accuracy of measurements. Therefore, such methods may not be sufficiently accurate to detect small changes (<1.5 kg) in FM or FFM, changes that would be considered clinically significant when making decisions about treatment of underweight patients with chronic gastrointestinal disease.

Dual-energy X-ray absorptiometry (DXA) is a precise, accurate, non-invasive, safe, and convenient technique, founded on a three compartment model separating the body into total body mineral mass (TBBMC), FM, and lean tissue mass (LTM), the latter being the remaining bone-free fat-free tissue mass [2,3]. In theory, DXA has the distinct advantage over most other body composition methods of not requiring any assumptions about the chemical constancy of the LTM, although it does assume constant attenuation of the lean and fat tissues. Thus, DXA may be an appealing option for body composition analysis in underweight patients with chronic gastrointestinal disease. However, the accuracy of DXA measurements in this particular group has only been sparsely documented [4-7]. Several factors may hypothetically affect the accuracy of DXA measurements in these patients, such as a low body weight per se, and an abnormal hydration status. In addition, it is unknown whether the constants and standard mathematical algorithms used for soft tissue determination are appropriate in this subgroup of patients.

The purpose of this study was twofold. Primarily, in underweight patients with short bowel syndrome dependent on long term home parenteral nutrition we evaluated whether DXA could accurately detect small changes in body composition induced by intravenous infusion of parenteral nutrition (PN). Secondly, to explore the influence of a low body weight per se on the accuracy of DXA, we investigated the ability of DXA to detect changes in body composition by placing packets of lard on the trunk and thighs of lean healthy volunteers.

## Methods

Two separate experiments were undertaken. In the hydration study, changes in body composition were induced by intravenous infusion of PN in underweight patients with short bowel syndrome. In the lard study, changes were induced by placing packets of lard on lean healthy volunteers.

### Anthropometry

All participants were weighed (after voiding) on a calibrated digital scale accurate within 0.1 kg (subjects wearing light clothes). The heights were measured after a maximal inhalation to the nearest 0.1 cm by using a wall-mounted stadiometer. The averages of two measurements for both height and weight were used as the criterion measurement. The body mass index was calculated as weight divided by height squared (kg/m^2^).

### Dual energy X-ray absorptiometry

Measurements of body composition were performed with the Norland XR-36 DXA densitometer (Norland Corporation, Fort Atkinson, WIS, U.S.A.) with the subject supine. The host software was rev. 2.5.2. and the scanner software rev. 2.0.0. The theory and methodology for body composition by DXA has previously been described [2]. Briefly, while the patient lightly dressed lay on a scan table for about 20 min., transverse scans approximately 1 cm apart were performed from top to heel. The instrument uses X-rays of two distinct energy levels that are attenuated by fat, bone and lean mass to different extent. By computerization of data inputs from approximately 11000 pixels DXA estimates body composition based on a three-compartment model, measuring TBBMC, FM, and LTM. The total mass (TM) by DXA is the sum of all three compartments, whereas the soft tissue mass (STM) by DXA includes only the LTM and FM. The fat free tissue mass (FFM) includes both the TBBMC and LTM. Among others, Hendel *et al*. [8] have reported precision errors of body composition of the Norland XR-36 densitometer. They were 2.2% for TBBMC, 2.7% for FFM, and 2.6% for FM%. In our hands the between-measurements CV%'s of TBBMC, LTM and FM, were 1.5%, 1.6%, and 3.9%, respectively [5].

### Hydration study

This study comprised 11 patients (9 women and 2 men) with short bowel syndrome treated with daily supplements of PN. The participants were selected for low body weight (BMI < 22 kg/m^2^). The diagnoses were: Crohn's disease (n = 6), ischaemic infarction (n = 2) and other (n = 3). Patients were on average (mean ± SD) 49.5 ± 17.1 yr., 1.58 ± 0.07 m, 48.5 ± 9.5 kg, and 19.3 ± 3.1 kg/m^2^. The PN was in all patients provided as a 3 L 'all in one' plastic bag containing a fixed composition of protein, glucose, and electrolytes, and four patients had additional supplements of 1–2 L of saline. The infusion was given continuously over a period of 8–10 hours during the night. Before starting the infusion all patients were weighed on a scale and scanned as described below. Immediately after completing the infusion the patients were reweighed and rescanned. Due to the large volume of intravenous fluid provided with the parenteral nutrition, patients were allowed to void during the study.

The theoretical soft-tissue attenuation (R_ST_) of an 'all in one' 3 L bag of PN was calculated using the equation R_ST _= Σ (-f_i _× (μ_mi_)_L_) / Σ (-f_i _× (μ_mi_)_H_), where (f_i_) is the mass fraction, (μ_mi_) is the mass attenuation coefficient of the i'th component at high (H) and low (L) photon energy levels [9]. The theoretical R_ST_-value for PN was calculated to 1.365, which is very close to that of normal saline (1.377). Given the calculated R_ST_-value PN should theoretically be scanned by DXA as consisting of approximately 2% FM and 98% LTM. Such values were confirmed in vivo by scanning one subject three times before and after placing 2 bags (6.96 kg) of PN on the subject's legs. By DXA, the equivalent 6.99 kg increase in TM (TM) resulted from a 7.31 kg gain of LTM (104.5%) and a 0.32 kg loss of FM (- 4.6%), whereas the TBBMC changed only 6 g (0.1%).

### Lard study

For this experiment two packets of porcine lard (with a small amount of muscle-tissue attached) were constructed and enclosed in plastic wrap. The lard packet dimensions were 19.8 cm × 38.8 cm × 2.6 cm, and weighed, using a beam scale, 3.49 kg. The total fat fraction of the lard, measured in triplicate by chemical fat extraction according to the method of Folch *et al. *[10], was 52.2% (CV% = 6.4%). The participants for this study were selected for low body weight (BMI < 22 kg/m^2^). Eight healthy lean male volunteers, who were on average (mean ± SD) 26.4 ± 7.4 years of age, agreed to participate. Anthropometric measures of the participants were taken immediately before the study and averaged 1.81 ± 0.07 m in height, 69.0 ± 7.7 kg in weight, and 21.0 ± 0.9 kg/m^2 ^in BMI. Without reposition, four consecutive total body DXA scans were performed on each participant. Two scans without added lard served as baseline measurements (the average values were used as the criterion measurements), and two scans were performed with the lard packets placed alternately on the abdomen centred over the lumbar vertebrae, and on the thighs at midpoint of the femur. The placements of the lard over the thighs and trunk were chosen to represent regions where the ability of DXA to correctly measure soft tissue composition is known to be good and poor, respectively.

### Ethics

The Ethics Committee for Medical Research in Copenhagen, Denmark, approved the study protocol and the study was conducted in accordance with the Declaration of Helsinki of 1975, as revised in 1983. Written and oral informed consent was obtained from all patients prior to inclusion.

### Statistics

All results are expressed as means ± standard deviation (SD) unless otherwise indicated. A paired Students t-test was used to compare paired variables. Association between variables was established by Pearson's correlation coefficients and linear regression. The CV%'s for the measurements of TBBMC or FM were calculated from the within-subjects SD's divided by their respective grand means. All statistical tests were two-tailed, and a *p *value of less than 0.05 was considered statistically significant. The SPSS statistical program version 10.0 (SPSS Inc., Chicago, USA) was used for all analyses.

## Results

### Hydration study

**Figure 1:**
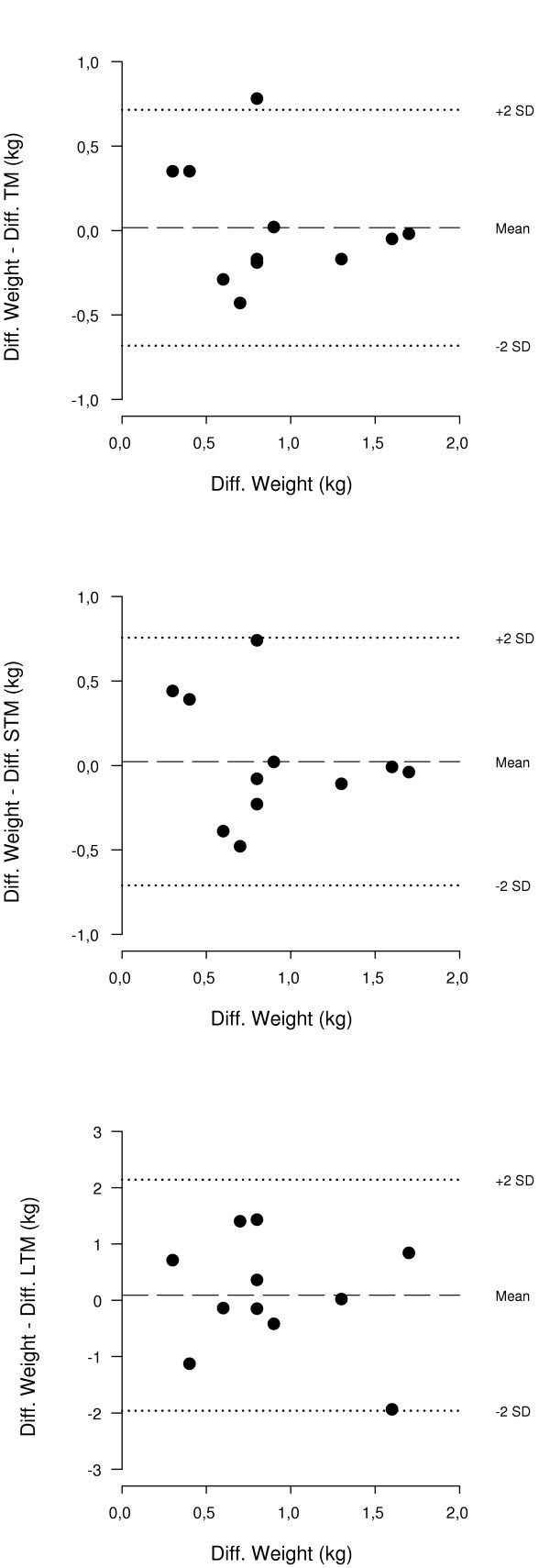
The figure displays the limits of agreement plots of the comparison of change in BW and changes in TM, STM and LTM by DXA following intravenous infusion of parenteral nutrition in 11 patients with short bowel syndrome.

The descriptive statistics for body weight (BW) and body composition variables by DXA before and after intravenous infusion of PN are given in Table 1. An average increase of 0.90 ± 0.45 kg of BW was achieved by infusion of PN. This corresponded to an increase in TM of 0.88 ± 0.63 kg, STM of 0.88 ± 0.64 kg, and LTM of 0.81 ± 1.20 kg which were significantly higher than the baseline values. No significant differences were found between baseline and post infusion estimates of FM and TBBMC. The average within subject CV%'s for TBBMC and FM were 2.0% and 3.5%, respectively (CV% for FM was expressed as the geometric mean, due to violation of the normality assumption). The correlations between changes in BW and changes in mass and composition by DXA are summarised in Table 2. The increase in BW correlated significantly to the increase in TM (R-square = 0.72, SEE 0.36 kg, p < 0.01), and the increase in STM (R-square = 0.69, SEE 0.38 kg, p < 0.01), however not with the increase LTM (R-square = 0.30, SEE 1.06 kg, p = 0.08). For all three regression lines (diff. BW vs. diff. TM, STM, and LTM) the intercepts were not significantly different from zero and the regression slopes were not significantly different from 1.00. Fig. 1 displays the limits of agreement plots of the comparison of change in BW and changes in TM, STM and LTM by DXA. The accuracy of DXA in the individual subject, expressed as the 95% confidence intervals for the difference between changes in BW and changes in DXA variables, was ± 2.06 kg for the detection of changes in LTM, and ± 0.71 kg for the detection of changes in STM and TM.

**Table 1:** Changes in body weight and body composition variables by DXA after infusion of parenteral nutrition in 11 patients on permanent home parenteral nutrition.

	Baseline	Post infusion	Change	Range
Body weight (kg)	48.39 ± 9.42	49.43 ± 9.20*	0.90 ± 0.45	0.30 ; 1.70
Total mass (kg)	47.91 ± 9.46	48.78 ± 9.06*	0.87 ± 0.65	-0.05 ; 1.72
Soft-tissue mass (kg)	45.88 ± 9.15	46.75 ± 8.75*	0.88 ± 0.65	-0.14 ; 1.74
Lean-tissue mass (kg)	32.51 ± 5.92	33.04 ± 5.33*	0.53 ± 1.36	-1.53 ; 3.54
Fat mass (kg)	13.37 ± 7.46	13.72 ± 7.82	0.35 ± 1.00	-1.93 ; 1.88
Total body bone mineral mass (kg)	2.03 ± 0.38	2.03 ± 0.39	0.00 ± 0.07	-0.10 ; 0.11

Values are mean ± SD. *Significantly different from baseline values (Students paired t-test)

**Table 2:** Intercorrelation of change in body weight and change in total mass, soft tissue mass, and lean tissue mass by DXA after infusion of parenteral supplements in 11 patients on permanent home parenteral nutrition.

	Body weight	Total mass	Soft-tissue mass
Total mass	0.846 *	-	-
Soft-tissue mass	0.832 *	0.994 *	-
Lean-tissue mass	0.550	0.497	0.471

* Correlations are significant (p < 0.01)

### Lard study

**Figure 2:**
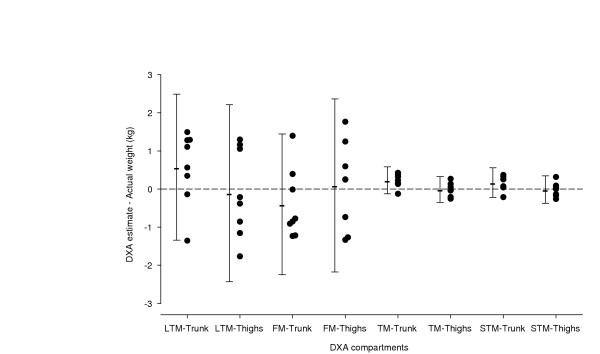
The figure shows the differences between the actual weight and composition of added lard and the measured values by DXA, in eight lean healthy volunteers. In each subject DXA measurements were performed with lard placement both on the trunk and on the thighs. Each symbol indicates measurements in one subject. Error bars are the 95% confidence intervals of the difference.

The placement of 3.49 kg of exogenous lard (with a composition of 52.2% FM and 47.8% LTM) over central and peripheral regions of the body of eight lean healthy volunteers had no effect on the estimate of TBBMC (p > 0.05, CV% = 1.4%). The descriptive statistics for the corresponding mean changes in TM, STM, LTM, and FM are given in Table 3. Except from a minor overestimation (0.23 kg) of TM and STM when the lard packets were placed over the trunk region, the measured changes in DXA variables were not significantly different from the expected values (p > 0.05, for all comparisons). However, DXA appeared slightly more accurate in detecting both the mass and composition of the added lard when the packets were placed over the thighs Table 3. Thus, whereas the composition into FM and LTM of the added lard placed over the thighs corresponded closely to the actual values, the FM was slightly underestimated and LTM correspondingly overestimated when the lard was placed over the trunk region. Fig. 2 shows the individual differences between the expected value of LTM, FM, TM and STM and the measured changes in the respective DXA variables. The accuracy of DXA in the individual subjects, expressed as the SD of the difference between expected and measured values, was 1.03 kg and 1.06 kg for the detection of changes in LTM and FM, respectively, and 0.18 kg for the detection of changes in STM and TM.

**Table 3:** The actual weight and chemical composition of lard packets placed on the thighs and abdomen of 8 healthy lean male volunteers and the composition measured by DXA.

		Thighs			Trunk		
	Actual	Mean ± SD	95% CI	Percentage Mass Detected Mean ± SD	Mean ± SD	95% CI	Percentage Mass Detected Mean ± SD

Total mass (kg)	3.49	3.48 ± 0.17	3.34 ; 3.62	99.7 ± 4.9	3.72 ± 0.18	3.57 ; 3.87	106.6 ± 5.1
Soft-tissue mass (kg)	3.49	3.48 ± 0.18	3.33 ; 3.63	99.6 ± 5.2	3.66 ± 0.19	3.50 ; 3.82	104.9 ± 5.5
Fat mass (kg)	1.82	1.92 ± 1.13	0.97 ; 2.86	105.2 ± 62.3	1.42 ± 0.92	0.65 ; 2.19	77.9 ± 50.8
Lean-tissue mass (kg)	1.67	1.56 ± 1.16	0.59 ; 2.53	93.5 ± 69.5	2.24 ± 0.96	1.44 ; 3.04	134.3 ± 57.3

## Discussion

Compared to several other body composition techniques DXA has a very high precision [8,11], which on paper should make DXA able to detect small changes in body composition. The precision errors, generally reported as the coefficient of variation (CV%) of repeated measurements, are about 2–3% for TBBMC, FM, and FFM in healthy subjects, and values of a quite similar proportion have been documented in underweight patients with chronic intestinal disease [5].

In addition to a high precision, DXA has proven an accurate method for body composition analysis in normal weight healthy subjects [12-17]. However, the accuracy in underweight patients with chronic gastrointestinal disease or in very lean subjects has only been sparsely elucidated [4,5,18], but may theoretically be lower due to factors inherent in the DXA methodology. DXA operates on a scanning principle separating the body into approximately 11 000 pixels of each 6.5 × 13.0 square mm, of which about 6000 pixels only contain soft tissue and about 5000 pixels contain both soft tissue and bone. Determination of total body composition of bone, fat, and lean tissue masses is based on computerised analysis of the soft tissue composition of each pixel separately. One important limitation of the DXA methodology is that direct estimation of soft tissue composition is possible only in pixels with no bone present. Evaluation of soft tissue composition in pixels with bone mineral as well as soft tissue is performed by extrapolating calculated values for soft tissue composition in adjacent bone-free pixels to the pixels with bone. In underweight patients with chronic gastrointestinal disease or other lean subjects, the number of none-bone containing pixels available is obviously reduced compared to normal weight subjects, and in theory, this may lower the accuracy of DXA. Additionally, in malnourished underweight patients, deviations in the state of hydration frequently occur, which might influence the accuracy of soft tissue determination by DXA. Thus, Pietrobelli et al. [19] demonstrated that fluctuations in the hydration affected soft tissue attenuation and gave rise to systematic and predictable errors in the determination of LTM and FM, although these errors were quite small with changes in hydration within normal physiological limits. Thus, simulated experiments showed that errors in the detection of FM% is <1% with hydration changes of 1–5% [19].

We studied underweight patients with gut failure due to short bowel syndrome, who were dependent on long-term home parenteral nutrition. The changes in TM, STM, and LTM of 0.90 kg induced by infusion of PN were accurately detected on a group level. Furthermore, measurements of TBBMC and FM, body constituents that should not be affected by changes in hydration, remained unchanged during the experiment, and the respective CV%'s were very close to normal precision errors for repeated measurements [5,8,11]. Our data agree with results of comparable experiments were changes in the hydration status of healthy volunteers were induced by intravenous saline infusion [15,16]. However, the accuracy errors (SEE's of the regression lines) of DXA in the individual patient were about 35% higher for the detection of TM and STM, and nearly 100% higher for the detection of LTM in our study compared to results in healthy subjects reported by Going *et al. *[16]. Yet, in the present study BW increased by only 0.90 kg compared to an increase of 1.21 kg in the study by Going *et al. *[16], a difference that may have affected the results.

To explore the influence of a low body weight per se on the accuracy of DXA, we investigated otherwise healthy lean young men (BMI = 21 kg/m^2^) with packets of lard placed over the trunk and thigh region. In agreement with previous studies in normal weight healthy volunteers [3,12-14,17] we found that the TM and STM of packets of lard were very accurately assessed by DXA regardless of position. In addition, the measured composition of the lard packets into FM and LTM was not significantly different from expected values with the packets overlying either the thigh or the trunk region. The accuracy error of DXA in lean subjects, expressed as the SD of the difference between expected and measured values, was about 1.04 kg for the detection of changes in LTM and FM, and 0.18 kg for the detection of changes in STM and TM. These values agreed closely with the reported accuracy errors for the Norland XR-36 scanner in normal weight healthy volunteers [17]. This indicates that a low body weight (BMI = 21 kg/m^2^) per se does not affect the accuracy of DXA. DXA appeared somewhat more accurate in detecting both the mass and composition of the added lard when the packets were placed over the thighs. Thus, the FM was slightly underestimated and the LTM correspondingly overestimated when the lard was placed over the trunk. Limitations in the ability of DXA to accurately assess the composition of soft tissue in the trunk region have been reported earlier. Thus, in common with our results Snead *et al. *[12] and Milliken *et al. *[13] reported that DXA underestimated FM of added lard placed on the trunk of healthy volunteers. This may be related to the fact that the trunk region contains a high degree of pixels with bone present because of the complex bone geometry in the trunk, which leaves relatively fewer bone-free pixels for the calculation of soft tissue composition in this region. Therefore, measurement of fat and lean may be less accurate in the trunk region compared to the extremities, which have more simple bone geometry and a relatively higher number of bone-free pixels.

We evaluated the performance of the Norland XR-36 (software version 2.5.2) densitometer to measure small changes in soft tissue composition in underweight patients with chronic gastrointestinal disease. DXA accurately detected changes in TM, STM, and LTM induced by infusion of PN on a group level, however the accuracy errors were up to 100% higher in this group compared to normal weight healthy subjects. Also, DXA performed well in detecting the composition of added lard placed on lean healthy subjects with accuracy errors similar to those reported in normal weight subjects, supporting that a low body weight per se does not affect the accuracy of DXA.

## Conclusions

We conclude that DXA is an accurate method for body composition analysis in underweight patients with chronic gastrointestinal disease. The individual accuracy errors however were higher than in normal weight subjects and this should be taken into account when evaluating the changes in body composition in the individual patient.

## Authors' contributions

KH and MS were responsible for conception and design of the study. KH, PHH and MS were responsible for data interpretation, and manuscript preparation. None of the authors have personal or financial interests in any organization sponsoring the research.
